# The critical role of Th17 cells and IL-17A in autoimmune and inflammation-associated neurological diseases: mechanisms and therapeutic perspectives

**DOI:** 10.3389/fimmu.2025.1656422

**Published:** 2025-11-20

**Authors:** Inmaculada Xu Lou, Huifen Zhou, Haitong Wan

**Affiliations:** 1 School of Basic Medical Sciences, Zhejiang Chinese Medical University, Hangzhou, China; 2 Zhejiang Key Laboratory of Chinese Medicine for Cardiovascular and Cerebrovascular Disease (2024JKZKTS19), Zhejiang, China; 3 Academy of Chinese Medical Sciences, Henan University of Chinese Medicine, Zhengzhou, Henan, China

**Keywords:** Th17, IL-17A, neuroinflammation, autoimmunity, RORγt, Treg

## Abstract

Helper T cells 17 (Th17) and their effector cytokine, interleukin-17A (IL-17A), play a dual role in immune homeostasis. On one hand, they are essential in defense against extracellular pathogens, such as bacteria and fungi, by inducing chemokine production and recruiting neutrophils. On the other hand, their dysregulated activity is strongly linked to autoimmune and inflammatory disorders, including multiple sclerosis, Alzheimer’s disease, Parkinson’s disease, and others. This article reviews the molecular mechanisms regulating Th17 differentiation and function, emphasizing the role of transcription factors like RORγt and RORα, as well as the influence of cytokines such as IL-6, IL-23, and TGF-β. Additionally, it explores the imbalance between pro-inflammatory Th17 cells and regulatory T cells (Tregs), a critical axis in the pathogenesis of autoimmune and neuroinflammatory diseases. In the context of neurological disorders, Th17 cells can infiltrate the central nervous system (CNS), where they contribute to neuroinflammation by activating microglia and astrocytes, exacerbating damage in conditions such as multiple sclerosis, traumatic brain injury, and neurodegenerative diseases. Emerging therapies, including anti-IL-17 monoclonal antibodies and natural modulators, are discussed as potential strategies to restore the Th17/Treg balance without compromising protective immunity. Finally, the need for further research is highlighted to elucidate the specific mechanisms of Th17 infiltration into the CNS, their interaction with the gut microbiota, and the development of personalized therapies. The integration of immunological, metabolic, and environmental approaches offers promising perspectives for the treatment of Th17/IL-17-mediated diseases.

## 1 Introduction

The IL-17 cytokine family comprises six structurally related proinflammatory cytokines, named IL-17A to IL-17F. These molecules are essential for host defense, promoting the expression of various cytokines and chemokines, facilitating neutrophil recruitment, influencing T cell differentiation, and enhancing the production of antimicrobial proteins. Maintaining an appropriate balance of IL-17 is essential for immune homeostasis; however, dysregulated production—particularly of IL-17A—is associated with the pathogenesis of numerous inflammatory and autoimmune diseases (1, 2).

Neuroinflammation, defined as a complex immune response within the CNS to insults such as infections, toxins, or injuries, involves the activation of resident cells (microglia and astrocytes) and the recruitment of peripheral immune cells. When sustained, this process becomes a common denominator in various neurological diseases, including neurodevelopmental disorders (e.g., autism spectrum disorders), neurodegenerative diseases (such as Alzheimer’s and Parkinson’s disease), and psychiatric conditions (including major depressive disorder) (3). Within this context, Th17 cells and their effector cytokine IL-17A have emerged as key mediators.

Recent studies have linked Th17/IL-17 to systemic autoimmune diseases (4), including their role in metabolic dysfunction—where an increase in pathogenic Th17 cells and a decrease in CD69+ T regulatory (Treg) cells exacerbate low-grade inflammation (5). For example, an imbalance between Th17 and Treg cells has been suggested as a potential biomarker for cardiovascular risk in individuals with rheumatoid arthritis (6). Moreover, IL-17A contributes to hypertension and target organ damage through mechanisms such as endothelial dysfunction, oxidative stress, and activation of proinflammatory pathways (NF-κB, MAPK) (7).

**Figure 1:**
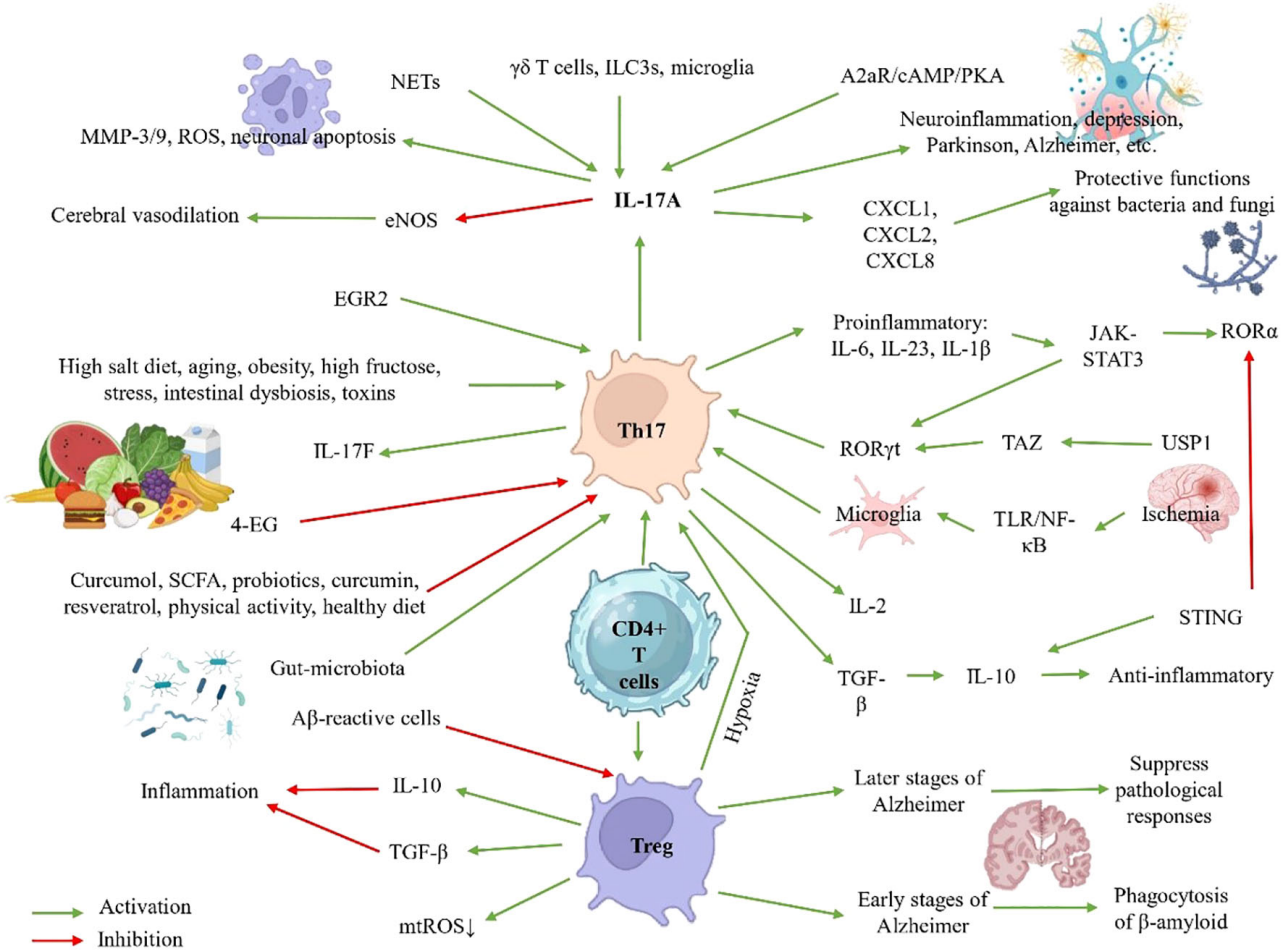
Th17 cell regulation in neuroinflammation: Pathways, Pathological Effects, and Protective Strategies.

Therapeutically, the blockade of IL-17A using monoclonal antibodies (such as secukinumab and ixekizumab) has demonstrated efficacy in diseases like psoriasis and ankylosing spondylitis (8), suggesting its potential applicability in neurological disorders mediated by neuroinflammation. Nevertheless, the specific role of Th17/IL-17 within the CNS remains an active area of research, particularly concerning their ability to cross the blood-brain barrier (BBB), activate microglia, and promote neuronal damage. Although not all neurological disorders are classified as neuroinflammatory, they often share a common hallmark: inflammation. This review aims to integrate current evidence regarding these mechanisms and their impact on brain diseases, with an emphasis on emerging therapeutic targets. **Figure 1** summarizes the key content of this article.

## 2 Search strategy and data sources

A systematic literature search was conducted in PubMed during October and November 2024 using a combined term strategy with Boolean operators. The search equation was: (“T Helper 17 Cell” OR “T Helper 17 Cells” OR “Type 17 Helper T Cells” OR “TH-17 Cells” OR “TH-17” OR “TH 17 Cells” OR “TH 17 Cell” OR “Th17 Cell” OR “Th17” OR “Type 17 Helper T Cell” OR “T helper 17 cells” OR “IL-17-producing T cells” OR “Pro-inflammatory T helper cells” OR “Type 17 T helper cells” OR “Th17 cells”). Alternative terms were deliberately excluded to minimize the risk of omitting relevant information. The inclusion criteria were: publications between January 2018 and November 2024, written in English, original and review studies, conducted in humans, animals, or *in vitro*, and directly related to the topic of this review. Exclusion criteria comprised case reports, conference abstracts, letters, comments, and congress proceedings. This methodology enabled the identification of relevant studies on the interaction between Th17 cells and immunity, particularly in inflammation-associated neurological disorders, through a two-stage selection process (title/abstract screening and full-text evaluation) to ensure the relevance of the included articles. A total of 12,521 articles were screened, of which 162 met the inclusion criteria and were incorporated into the review.

## 3 Biology and functional roles of Th17 cells

### 3.1 Differentiation and regulation

The differentiation of Th17 cells is a complex process orchestrated by multiple cytokine signals and molecular transduction pathways. Key factors include IL-6, IL-23, IL-1β, TGF-β, and IL-21, which activate the JAK-STAT3 pathway and induce the expression of the transcription factors RORγt and RORα, fundamental determinants of the Th17 lineage. Interestingly, the functional profile of these cells—whether pathogenic or protective—depends on the cytokine context: while IL-6, IL-23, and IL-1β promote a proinflammatory phenotype, TGF-β favors IL-10 production, conferring anti-inflammatory properties. IL-17A, considered the hallmark cytokine of Th17 cells, is also produced by other immune populations such as γδ T cells, ILC3s (type 3 innate lymphoid cells), and microglia, further amplifying its impact on inflammatory responses (9).

At the transcriptional level, the maintenance of the Th17 effector program depends on the cooperative activity of RORγt and RORα, which engage cis-regulatory elements within the RORC locus. RORα deficiency leads to diminished RORγt expression and impaired Th17 effector function, emphasizing its critical role in maintaining lineage stability (10, 11). Additional factors, such as STAT3—crucial for Th17 differentiation—promote the activation of miR-384, a microRNA that enhances RORγt expression and IL-17 secretion (12). Moreover, metabolic factors such as the LKB1-PTEN pathway modulate Th17 differentiation via mTORC1-HIF1α, where LKB1 deficiency enhances glycolysis and favors an inflammatory phenotype (13).

The plasticity of Th17 cells is another critical aspect, particularly at mucosal barriers, where they can adopt pro- or anti-inflammatory profiles depending on microenvironmental signals (14). This adaptability is finely regulated by intrinsic mechanisms such as STING (Stimulator of Interferon Genes), an inhibitory checkpoint that induces IL-10 and reduces RORγt expression in non-pathogenic Th17 cells (15, 16), and by extrinsic factors such as IGF-1, which promotes pathogenicity through Akt-mTOR activation and enhanced aerobic glycolysis (17).

Dysregulation of these processes has significant pathological consequences. In autoimmune diseases, pathogenic Th17 cells display enhanced glycolytic activity, regulated by the miR-21/Peli1-c-Rel axis (18), along with abnormal ubiquitination of RORγt mediated by USP19 (19). Additionally, signals such as adenosine (via the A2aR/cAMP/PKA axis) (20) and components of neutrophil extracellular traps (NETs) (via TLR2/MyD88) (21) enhance IL-17 secretion, further exacerbating inflammation.

Emerging therapeutic strategies aim to restore the Th17/Treg balance, either through RORα antagonists (22), inhibition of ADAM9 (A Disintegrin And Metalloproteinase 9)—a metalloproteinase that activates TGF-β1 (23)—or modulation of Foxo1, a key regulator of lipid metabolism in Th17 cells (24).

### 3.2 Cytokine secretion profile

Th17 cells are defined by their production of a characteristic array of proinflammatory cytokines, with IL-17 family members—especially IL-17A and IL-17F—being particularly prominent. Although these molecules are structurally related, they exhibit significant differences in their regulation, potency, and physiological roles (25, 26). The differentiation of Th17 cells in humans is tightly regulated by a specific cytokine milieu that includes TGF-β, IL-6, IL-1β, and IL-21, while IL-23 plays a crucial role in the maintenance and survival of this cell lineage. Once differentiated, Th17 cells exert their inflammatory effects primarily through the secretion of IL-17A and IL-17F, which, although sharing similar mechanisms of action (e.g., induction of IL-6, IL-8, and CXCL1 in fibroblasts and epithelial cells), differ in their biological potency, with IL-17A being considerably more potent. Both cytokines have the capacity to markedly amplify their inflammatory potential by acting synergistically with other cytokines such as TNF-α, thereby creating a microenvironment that perpetuates the inflammatory response (25, 27, 28).

## 4 Th17 cells in host defense and autoimmunity

### 4.1 Protective and pathogenic roles

Th17 cells are critical effectors of the adaptive immune system, playing a central role in host defense against extracellular pathogens, particularly bacteria and fungi. Their activity is mediated through the induction of proinflammatory signals, recruitment of effector immune cells, and enhancement of antimicrobial factor expression, ultimately promoting pathogen clearance. A key driver of these responses is the cytokine IL-17A, which strongly induces the expression of chemokines (CXCL1, CXCL2, CXCL8) and cytokines that mediate the recruitment and activation of neutrophils and other myeloid cells at sites of infection or tissue injury. In parallel, cytokines such as G-CSF and IL-6 amplify innate inflammation by promoting myeloid cell–mediated responses (9).

The relevance of Th17 cells in immunity becomes evident in situations of deficiency in this subpopulation, which can manifest as increased susceptibility to recurrent infections, particularly fungal infections. This condition should be considered in the diagnosis of patients with persistent fungal infections, as impaired Th17 function compromises host defense against extracellular and intracellular microorganisms, parasites, fungi, and viruses (29). Additionally, recent studies have demonstrated that increased extracellular sodium activates Th17 cells, enhancing their capacity to protect the organism against bacterial and fungal infections. IL-17, produced by both Th17 cells and CD8+ T lymphocytes (Tc17), is critical for immune responses at epithelial surfaces. However, its role is not limited to protection, as it has also been associated with autoimmune diseases. Inherited defects in IL-17-mediated immunity can lead to chronic mucocutaneous candidiasis, a disorder characterized by bacterial and fungal infections of the mucous membranes, often accompanied by clinical manifestations of immune dysregulation (30).

CD4+ T cells are essential components of the adaptive immune system, with specialized subpopulations including Th1, Th2, Th17, Treg, and Tfh cells. While Th1 cells act against intracellular pathogens and Th2 cells target helminths, Th17 cells are fundamental for the response against extracellular bacteria and fungi. Their differentiation is regulated by cytokines such as IL-6 and IL-23, and their main transcription factor, RORγt, is crucial for their development and function. Additionally, ILC3s share similar effector functions with Th17 cells. The plasticity of Th17 cells is another relevant aspect, as they can transdifferentiate into a Th1-like phenotype in response to microenvironmental factors, such as changes in the microbiota or angiogenic signals. This versatility is linked to their metabolic heterogeneity and may influence their involvement in both protective responses and autoimmune pathologies. Genetic alterations in key components of the Th17 pathway, such as mutations in RORC or STAT3, are associated with diseases like chronic candidiasis or hyper-IgE syndrome. Similarly, defects in cytokines such as IL-1β affect Th17 differentiation and are related to autoimmune and inflammatory disorders (31).

Since their discovery in 2005, Th17 cells have been recognized as a double-edged sword in immunity. While indispensable for defense against extracellular pathogens, their dysregulated activity and proinflammatory mediators (IL-17, TNF-α) drive tissue damage and chronic inflammation. Accordingly, Th17 cells can be classified into non-pathogenic and pathogenic subsets, with the latter contributing to autoimmunity. Recent findings highlight the role of microRNAs in modulating Th17 pathogenicity, thereby influencing their capacity to trigger autoimmune responses (9, 32).

### 4.2 Interaction with treg cells

Th17 and Treg cells are two distinct CD4+ T lymphocyte subsets that play opposing roles in the regulation of immune responses. While Th17 cells promote inflammation and are implicated in the pathogenesis of autoimmune diseases, Tregs exert suppressive effects that maintain immune homeostasis (33). This dynamic balance is crucial for an appropriate immune response, as its dysregulation can lead to chronic inflammatory diseases or immunosuppression. Th17 cells have the ability to cross the BBB, where they trigger inflammatory cascades and recruit other immune cells into the CNS. In contrast, Tregs modulate these responses by releasing inhibitory cytokines such as IL-10 and TGF-β, as well as through contact-dependent suppression mechanisms (34).

The differentiation of naïve CD4+ T cells into Th17 or Treg lineages is finely regulated by intracellular signals, including T cell receptor (TCR) pathways and ITK kinase, which activate MAPK cascades and calcium signaling (35). Within the CNS, Tregs interact with resident cells such as microglia and astrocytes, modulating neurodegenerative processes. In early stages of Alzheimer’s disease (AD), Tregs promote the phagocytosis of β-amyloid peptides by microglia, whereas at later stages, they suppress pathological responses mediated by Th1 and Th17 cells (36). Other cellular subtypes, such as Th9 cells, also influence this balance through the production of IL-9, which can modulate the Th17/Treg ratio under conditions like experimental cerebral malaria (37).

Microenvironmental factors such as hypoxia play a key role in the polarization of these populations. In immune thrombocytopenia, hypoxic conditions promote the expression of HIF1α, which disrupts the Treg/Th17 balance (38). Moreover, chronic hypoxic conditions can promote the transdifferentiation of Tregs into Th17 cells, favoring a proinflammatory environment associated with pulmonary hypertension (39). Conversely, adenosine exerts immunomodulatory effects by promoting the development of IL-17+ regulatory cells, although in murine models it predominantly promotes Treg expansion (40).

Therapeutically, strategies aimed at modulating this balance show great potential. Mesenchymal stem cells can induce an anti-inflammatory phenotype in macrophages, enhancing Treg differentiation while reducing the Th17 proportion (41). At the molecular level, the enzyme USP1 regulates the stability of TAZ, a co-activator of RORγt that promotes Th17 differentiation while inducing the degradation of Foxp3, a key protein for Treg identity. USP1 inhibitors, such as ML323, have shown the ability to restore this balance in autoimmune disease models (42, 43). Similarly, the Akt/mTORC1 pathway, activated by CK2, favors Th17 polarization through HIF1α and RORγt, while suppressing Treg differentiation. The inhibitor CX4945 (Silmitasertib) reverses these effects, promoting Foxp3 expression (44).

In pathological contexts such as MS, mitochondrial oxidative stress in Tregs can impair their suppressive function, allowing Th1- and Th17-mediated autoimmune responses. Reducing mitochondrial reactive oxygen species (mtROS) restores Treg activity and attenuates neuroinflammation (45). These findings underscore the critical importance of the Th17/Treg balance in neurological disorders, where, despite the greater infiltrative capacity of Th17 cells across the BBB, Tregs are essential for limiting their pathogenic activity (46). Overall, the interaction between Th17 and Treg cells constitutes a central axis in the immunopathology of various diseases.

## 5 Th17 cells and neuroinflammation

### 5.1 Mechanisms of neuroinflammation

Neuroinflammation is a complex process in which Th17 cells play a central role, particularly in autoimmune diseases of the CNS (47). The transcription factor EGR2 (Early Growth Response 2) emerges as a key regulator of Th17 pathogenicity and migration to the CNS. In patients with MS and in animal models of neuroinflammation, EGR2 is overexpressed in myelin-reactive T cells, where it reinforces the Th17 transcriptional program in a RORγt-dependent manner. EGR2 is not essential for basic Th17 differentiation but is critical for their ability to recruit myeloid cells to the CNS and trigger neuroinflammation, without compromising host immunity against infections. This mechanism explains how Th17 cells acquire CNS-specific pathogenic properties (48).

The interaction between dendritic cells and Th17 cells is another critical axis in neuroinflammation. The formyl peptide receptor 2 (FPR2) in dendritic cells regulates their metabolism and nitric oxide production, which in turn modulates Th17 polarization. FPR2 deficiency disrupts this metabolic balance, increasing nitric oxide and reducing Th17 differentiation, thereby attenuating autoimmune encephalomyelitis (49). Additionally, the transcription factor TCF4 in dendritic cells exerts a protective effect by limiting the uncontrolled differentiation of Th1 and Th17 cells during neuroinflammation (50).

The migration of Th17 cells into the CNS is tightly regulated by adhesion molecules such as integrin α3, which promotes the formation of immunological synapses, Th17 proliferation, and their extravasation across the BBB. Deletion of integrin α3 in Th17 cells reduces the severity of encephalomyelitis by preventing their migration into the brain parenchyma, retaining them in the perivascular space (51). Interestingly, Th17 cells preferentially cross the cerebrospinal fluid–brain barrier (BCSFB) under both inflammatory and non-inflammatory conditions, a process mediated by ICAM-1 (52).

### 5.2 Experimental autoimmune encephalomyelitis and multiple sclerosis

Multiple sclerosis (MS) and its animal model, experimental autoimmune encephalomyelitis (EAE), represent paradigms of neuroinflammatory diseases mediated by the infiltration of autoreactive T cells into the CNS. This process triggers an inflammatory cascade that results in demyelination, axonal damage, and neurodegeneration. Among the key cell populations, Th17 cells emerge as central players in pathogenesis, although their interaction with other immune components reveals a complex network of regulation (53–55).

Th17 cells exhibit remarkable plasticity and the ability to infiltrate the CNS, processes regulated by multiple molecular pathways. The integrin α3, highly expressed on Th17 cells in the CNS during EAE, facilitates their migration by promoting the formation of immunological synapses and extravasation through the BBB (51). Notably, these cells also exhibit intestinal tropism: in EAE models, myelin-specific Th17 cells infiltrate the lamina propria of the colon prior to the appearance of neurological symptoms, altering the gut microbiota. Blocking their intestinal entry with anti-α4β7 antibodies attenuates EAE, suggesting an intestinal-brain axis in pathogenesis (56).

At the metabolic level, mitochondrial oxidative phosphorylation (OXPHOS) is essential for Th17 pathogenicity as it sustains the activation of BATF (basic leucine zipper transcription factor TF-like), a critical transcription factor for their differentiation (57). Additionally, acetylcholine transferase in Th17 cells, negatively regulated by TGF-β but induced by strong TCR signals, promotes chronic activation through acetylcholine production, exacerbating EAE (58). Several mechanisms regulate the differentiation and function of Th17 cells. C-reactive protein indirectly inhibits Th17 responses by influencing antigen presentation by dendritic cells (moDCs) via FcgR2B, thereby suppressing NF-κB and ERK signaling pathways (59). On the other hand, cathelicidin, an antimicrobial peptide produced by neutrophils and microglia, enhances Th17 differentiation and their transition to IFN-γ producing phenotypes, aggravating EAE (60). The ubiquitin ligase Cbl-b plays a crucial regulatory role by suppressing IL-6 production in macrophages, thereby restricting the polarization of pathogenic Th17 cells (61). Meanwhile, the methyltransferase METTL3 regulates Th17 differentiation via m6A modifications on RNA, and its deficiency attenuates EAE (62).

Dietary and pharmacological interventions show therapeutic potential. 4-ethylguaiacol, a phenolic compound found in the diet, inhibits microglial activation, protects the BBB, and reduces Th1/Th17 cell infiltration within the CNS (63). AdMSCs (adipose-derived mesenchymal stem cells) modulate the Th17/Treg balance, increasing anti-inflammatory cytokines such as IL-27 and IL-33 (64). Modulation of neurotransmitters also offers innovative perspectives. Inhibition of acetylcholine through vagotomy reduces the severity of EAE by suppressing Th17 proliferation and favoring Th2 responses (65).

### 5.3 Traumatic brain injury

Traumatic brain injury (TBI) is one of the leading causes of disability and mortality worldwide, with secondary damage resulting from neuroinflammation playing a critical role in neurological outcomes. While the primary damage caused by mechanical trauma is irreversible, the subsequent immune response—characterized by an imbalance between pro-inflammatory (e.g., Th17) and regulatory (Treg) lymphocyte populations—largely determines functional recovery. Recent studies have demonstrated that transplantation of umbilical cord-derived mesenchymal stem cells (UCMSCs) can modulate this balance through the TGF-β/Smad3/NF-κB pathway, thereby reducing neuroinflammation and improving cognitive function in animal models. This effect is attributed to the ability of UCMSCs to suppress Th17 polarization while promoting Treg expansion, thus mitigating secondary injury and favoring neurological repair (66).

A particularly relevant aspect of TBI pathophysiology is the role of Th17-type responses in exacerbating long-term neuronal damage. IL-17-producing CD4+ T cells not only facilitate the migration of immune cells across the BBB but also enhance the cytotoxic activity of CD8+ lymphocytes by inducing granzyme B and perforin production. In murine models, this synergy between Th17 and CD8+ cells is associated with persistent neuronal damage, myelin pathology, and prolonged neurological impairment. Experimental depletion of CD8+ T cells confers protection against these sequelae, highlighting the pathogenic role of this immune axis in TBI progression (67). In cases of severe TBI, immune dysregulation is even more pronounced, with excessive production of pro-inflammatory cytokines (IL-1β, IL-6, TNF-α) by activated microglia and infiltrating T cells, where Th17 cells significantly contribute to tissue damage in contrast to the protective effect mediated by Tregs (68). These findings reveal a dual paradigm in the role of the immune system following TBI: while Th17 and CD8+ responses exacerbate neurological damage through cytotoxic and pro-inflammatory mechanisms, strategies that promote a shift toward immunoregulation—such as UCMSCs therapy or Treg expansion—represent promising therapeutic approaches.

### 5.4 Parkinson’s disease

Parkinson’s disease (PD) is a complex neurodegenerative disorder where the progressive loss of dopaminergic neurons in the substantia nigra is associated not only with Lewy body pathology but also with an active immune response that amplifies neuronal damage. Recent studies highlight the imbalance between pro-inflammatory Th17 cells and regulatory Tregs as a key mechanism in the peripheral and central neuroinflammation that characterizes this disease (69, 70). Th17 cells, considered one of the most harmful lymphocytes in PD, exert their pathogenic effects primarily through the secretion of IL-17, which stimulates the production of additional pro-inflammatory molecules, including IL-1β and TNF-α. These cytokines activate apoptotic signaling pathways by binding to specific receptors on dopaminergic neurons, accelerating their degeneration, especially in the early stages of the disease (71).

Experimental evidence demonstrates that this Th17/Treg imbalance is not merely an epiphenomenon but an active factor in pathogenesis. While Th17 cells promote the death of nigrostriatal neurons, Tregs attenuate these neurotoxic effects, suggesting that the ratio between these populations may determine the progression of neurodegeneration (69). This paradigm has driven the development of innovative therapeutic strategies, such as the use of AdMSCs, which modulate the immune response by suppressing effector T cells and have shown potential in treating immune-mediated disorders. Similarly, preparations like DiHuangYin decoction have demonstrated neuroprotective effects in murine models of MPTP-induced PD, significantly reducing IL-17A levels in serum and the frequency of Th17 cells in the spleen, correlating with motor improvement and preservation of dopaminergic neurons (72).

At the molecular level, proteins such as JKAP emerge as critical regulators of this immune axis. In PD patients, JKAP is decreased and shows a negative correlation with Th1 and Th17 responses. *In vitro* experiments reveal that its overexpression inhibits the activation and differentiation of CD4+ T cells toward Th1/Th17 phenotypes, while its silencing has the opposite effect, suggesting that restoring JKAP could be a promising strategy to rebalance the immune response in PD (73). Additionally, studies in animal models highlight how Th17 stimulation with α-synuclein, a key protein in PD, exacerbates neuronal death in the substantia nigra, reinforcing the role of these cells in the neurodegenerative cascade (9).

### 5.5 Alzheimer’s disease

Alzheimer’s disease (AD) represents a complex neurodegenerative challenge where neuroinflammation driven by the immune system plays a central pathogenic role. Recent studies reveal that effector CD4+ T cells, particularly Th1 and Th17 subtypes, accelerate the progression of AD by disrupting the peripheral and central immune balance. In murine models, these amyloid-β (Aβ)-reactive cells have been shown to exacerbate pathology by suppressing the activity of Tregs both peripherally and within the CNS, thereby promoting Aβ accumulation and tau aggregation (74). The cytokine IL-17, predominantly produced by Th17 cells, emerges as a key mediator in this process. Experimental evidence demonstrates that neutralization of IL-17 not only rescues Aβ-induced cognitive deficits but also attenuates systemic inflammation, vascular dysfunction, and the prothrombotic state associated with AD, revealing the multifaceted role of this pathway in pathogenesis (75).

At the molecular level, alterations in immune regulators such as JKAP (JNK-associated phosphatase) correlate with the progression of cognitive decline in AD patients. JKAP, which modulates the differentiation of CD4+ T cells, is decreased in AD, while Th17 populations are elevated, creating a pro-inflammatory environment that exacerbates synaptic dysfunction and phosphorylated tau pathology (76). This Th17/Treg imbalance is also influenced by metabolic factors such as 27-hydroxycholesterol, whose elevated levels in patients with mild cognitive impairment promote polarization toward Th17, increase Aβ accumulation, and worsen cognitive performance. Inhibition of RORγt, a key transcription factor for Th17, reverses these effects, highlighting its therapeutic potential (77).

Immunomodulatory strategies have shown promising results. Low-dose IL-2 treatment in the mid-stages of AD restores the Treg/Th17 balance, reduces neuroinflammation, and decreases amyloid plaque burden in APP/PS1 (Amyloid Precursor Protein/Presenilin-1) models, significantly improving cognition (78). Furthermore, *in vitro* studies and animal models have demonstrated that IL-17A fosters pathological neuronal autophagy and neurodegeneration. The disruption of the BBB in AD facilitates the infiltration of neutrophils and Th17 cells into the brain parenchyma, sustaining a cycle of IL-17A production and neuronal injury. Administration of anti-IL-17A antibodies mitigates these effects, reducing neuroinflammation and improving cognitive function (9).

### 5.6 Epilepsy

Epilepsy is one of the most prevalent neurological disorders, affecting approximately 0.5–1% of the global population, with a lifetime incidence of 1–3%. Although pharmacological therapy remains the first-line treatment, nearly 30% of patients continue to experience seizures (79). Increasing evidence supports that neuroinflammation plays a central role in epileptogenesis and disease progression. Epileptic activity triggers the release of proinflammatory mediators such as IL-1β, IL-6, TNF-α, CCL2, ROS, iNOS, and IFN-γ, leading to neuronal damage, reduced synaptic plasticity, cognitive impairment, and psychiatric comorbidities. Among these mediators, IL-17 has emerged as a key contributor to epileptic pathology (79, 80).

IL-17 promotes peripheral immune cell infiltration and impairs GABAergic inhibitory transmission, thereby enhancing neuronal excitability (79). Studies in rat models of epilepsy demonstrated that IL-17 accumulates in the brain and induces neuronal hypersensitivity, necrosis, and neurocognitive dysfunction. In patients with epilepsy, IL-17 expression is elevated in epileptogenic lesions and serum, correlating with neuronal hyperexcitability, dysfunction, and cell death (81, 82). Likewise, γδ T cells producing IL-17 and GM-CSF have been shown to accumulate in epileptogenic foci, with their abundance positively associated with disease severity (82).

The Th17/Treg balance is essential for immune homeostasis in the brain. While Th17 cells are proinflammatory and neurotoxic, Treg (CD4^+^Foxp3^+^) lymphocytes exert immunosuppressive effects that limit excessive inflammation and maintain tolerance (83). In epileptic conditions, this balance shifts toward Th17 predominance, as reflected by decreased Treg frequency and function, reduced FoxP3 expression, and upregulated RORγt-mediated Th17 differentiation. Clinical and experimental studies indicate that Treg depletion exacerbates seizure severity, whereas restoring Th17/Treg equilibrium alleviates disease symptoms (79).

Furthermore, drug-resistant epilepsy has been linked to an expansion of Th17 and Th1 subsets, suggesting that adaptive immune dysregulation contributes to pharmacoresistance (84). Patients with focal epilepsy of unknown cause exhibit increased Th17 cell ratios and diminished regulatory lymphocyte populations, reinforcing the role of adaptive immunity in epileptogenesis. Therapeutically, interventions such as ketogenic diet have been proposed to modulate Th17/Treg balance by inhibiting the mTOR/HIF-1α pathway and enhancing anti-inflammatory mediators (IL-10, IL-4, GATA3) (79). Additionally, probiotics and SCFAs (short-chain fatty acids) have shown potential to suppress IL-17 signaling, restore intestinal homeostasis, and increase seizure threshold in experimental models (82).

## 6 Th17 cells and cerebrovascular pathologies

### 6.1 Ischemic stroke

Ischemic stroke represents one of the leading causes of mortality and disability worldwide, with the inflammatory response playing a dual role in its pathophysiology. On one hand, neuroinflammation exacerbates secondary brain damage in the early stages; on the other hand, it favors repair processes in the later stages. Th17 cells and their effector cytokine, IL-17A, emerge as central players in this process, not only exacerbating ischemic injury but also contributing to long-term complications such as cognitive decline (85, 86).

After a stroke, the interaction between microglia and peripheral T lymphocytes establishes a vicious circle of neuroinflammation. Microglia, activated via TLR/NF-κB minutes after ischemia, recruit T cells to the CNS through antigen presentation. These cells, in turn, promote microglia polarization towards a proinflammatory phenotype, perpetuating the damaging response (87). Th17 cells, which reach their peak peripheral levels 3–5 days post-stroke, infiltrate the ischemic tissue through a disrupted BBB, where they release IL-17A. This cytokine amplifies the damage through multiple mechanisms (1): increases BBB permeability by inducing the expression of metalloproteinases (MMP-3/9) (2); recruits neutrophils that release reactive oxygen species (ROS) (3); promotes neuronal apoptosis by regulating proteins such as caspase-3 and BAX (Bcl2 Associated X Protein); and (4) inhibits cerebral vasodilation by reducing endothelial nitric oxide (eNOS) production (85).

External factors, such as diet, modulate this response. High salt intake activates the p38/MAPK pathway in Th17 cells, increasing IL-17A levels and exacerbating cerebrovascular dysfunction (85). Conversely, SCFAs derived from the gut microbiota exert protective effects by reducing γδT+IL-17+ cells in the brain (85).

Clinically, the concentrations of Th17 cells and IL-17A in peripheral blood are associated with stroke severity and serve as predictors of neuropsychiatric complications. Patients with persistently elevated IL-17A levels show higher risks of cognitive decline, anxiety, and depression, as evidenced by lower scores on the Mini-Mental State Examination (MMSE) (88, 89). The protein RBP4 emerges as a promising marker, as its increase is associated with Th17/Treg imbalance and predicts cognitive decline at 3 years (90).

Modulation of the Th17/Treg axis offers therapeutic opportunities. The compound 2-(-2-benzofuranyl)-2-imidazoline reduces infarct volume and improves neurological deficits by suppressing Th17 and elevating Tregs, normalizing IL-17A and IL-10 levels (91). Another strategy involves myeloid-derived suppressor cells (MDSCs), whose adoptive transfer attenuates ischemic injury by inhibiting glycolysis and limiting differentiation towards Th1/Th17 (92). The phosphatase JKAP, which correlates with lower Th17 percentages, emerges as another potential target (93). Aging, a key risk factor for stroke, exacerbates brain inflammation by increasing proinflammatory populations such as Th17 (94).

### 6.2 Intracerebral hemorrhage and subarachnoid hemorrhage

Intracerebral hemorrhage (ICH) and subarachnoid hemorrhage (SAH) represent neurological emergencies with high rates of mortality and disability, where the secondary immune-inflammatory response significantly amplifies the initial neurological damage. In SAH, particularly in cases associated with intracranial aneurysm rupture, the balance between pro-inflammatory Th17 cells and regulatory Tregs emerges as a key determinant in the progression of injury. Recent studies demonstrate that dysregulation of this axis favors a harmful neuroinflammatory microenvironment, exacerbating neurological sequelae (95).

At the molecular level, Toll-like receptors (TLRs) play a fundamental role in modulating this immune response. Activation of TLR4 and TLR2 after ICH induces dendritic cell maturation, leading to an immune imbalance characterized by a decrease in Tregs and a relative increase in Th17 cells. This phenomenon is associated with exacerbated neuroinflammation and worse neurological outcomes. Interestingly, TLR9 exerts opposite effects: although it also promotes dendritic cell maturation, it stabilizes the Treg/Th17 ratio and attenuates inflammation. These receptors operate through distinct signaling pathways: TLR4 utilizes the p38-MAPK/MyD88 pathway, while TLR9 operates via IDO1/GCN2, highlighting the complexity of these immune-regulatory networks (96).

The clinical implications of these findings are profound. First, they suggest that selective modulation of specific TLR pathways could represent a therapeutic strategy to rebalance the Th17/Treg response after brain hemorrhage. Second, they highlight the central role of dendritic cells as a bridge between TLR activation and adaptive T cell responses, positioning them as promising therapeutic targets. Finally, these mechanisms explain why patients with similar hemorrhagic patterns may exhibit divergent clinical outcomes depending on their immune activation profile (96).

### 6.3 Atherosclerosis and vascular inflammation

Atherosclerosis, the leading cause of cardiovascular disease, is a chronic inflammatory process characterized by an imbalance between Th17 (pro-inflammatory) and Treg (regulatory) lymphocyte subsets. Recent studies have shown that the phosphatase JKAP acts as a key regulator of this balance, where its overexpression suppresses Th1/Th17 differentiation and reduces the production of pro-inflammatory cytokines, while its deficiency in CD4+ lymphocytes exacerbates the formation of atherosclerotic lesions by activating ERK and NF-κB pathways in the aorta (97). Concurrently, the NLRP3 inflammasome has emerged as another critical modulator, with elevated serum levels of NLRP3 negatively correlating with the Treg/Th17 ratio in patients with obstructive coronary artery disease, promoting the release of IL-1β, IL-6, and IL-17, thereby perpetuating vascular inflammation (98).

Th17 cells, through IL-17A, accelerate plaque progression through multiple mechanisms: they induce the expression of adhesion molecules (VCAM-1) on vascular smooth muscle cells (VSMCs) via NF-κB, facilitating leukocyte recruitment; increase the production of metalloproteinases (MMP-9) and chemokines (MCP-1) in macrophages, which degrade the extracellular matrix; and enhance the internalization of oxidized LDL (oxLDL) by regulating the LOX-1 receptor in macrophages (99).

Environmental factors, such as a high-salt diet, exacerbate this process by promoting a highly pathogenic Th17 phenotype (expressing GM-CSF, IL-23R, and CCR6) and reducing Treg function (100). Regarding therapeutic interventions, statins have been shown to improve the Th17/Treg balance in coronary patients by reducing IL-17/IL-6 and increasing IL-10/TGF-β, while pioglitazone stabilizes plaques by phosphorylating key proteins and regulating RORγt/Foxp3 (100). Furthermore, losartan mitigates uremia-induced atherosclerosis by regulating the Treg/Th17 balance via the PTEN/PI3K/Akt signaling pathway (101). Natural alternatives such as resveratrol and curcumin have also shown beneficial effects by modulating the intestinal microbiota, reducing levels of the pro-atherogenic metabolite TMAO, and restoring the Treg/Th17 balance (102). The discovery of novel biomarkers, such as circulating Th17 levels (which correlate with carotid stenosis severity), and therapeutic targets like TRIM21—which modulates Th17 responses and stabilizes plaques through fibrosis—provides new opportunities for the development of personalized therapies (103, 104).

## 7 Therapeutic modulation of Th17 cells

### 7.1 Natural compounds and probiotics

Natural compounds and probiotics have emerged as promising strategies to modulate the immune response mediated by Th17 cells, offering therapeutic benefits in autoimmune, neurodegenerative, and metabolic diseases. Curcumol, a compound derived from turmeric, has shown efficacy in reducing neuroinflammation after cerebral ischemia-reperfusion injury. This effect is achieved by polarizing microglia towards an anti-inflammatory phenotype and restoring the Treg/Th17 balance, modulating the Nrf2/HO-1 and NF-κB pathways. Additionally, curcumol decreases the production of ROS and improves motor recovery in animal models, highlighting its neuroprotective potential (33).

In the context of PD, the gut microbiota plays a key role in influencing Th17 differentiation, suggesting that microbiome-targeted interventions could modulate neuroinflammation and motor symptoms (9). Similarly, the Huangqi-Guizhi-Wuwu decoction regulates T cell differentiation in EAE, increasing Tregs and suppressing Th1 and Th17 by inhibiting JAK/STAT pathways (105).

SCFAs, such as propionic acid, exert immunomodulatory effects by reducing neuroinflammation and improving cognitive function in perioperative neurocognitive disorders. These microbial metabolites regulate Th17 responses, reinforcing the gut-brain axis and immunity connection (106). On the other hand, the Angong Niuhuang Pill shows anti-atherosclerotic effects by modulating the Th17/Treg balance and reducing chronic inflammation in atherosclerosis models (107).

Probiotics, particularly strains of *Lactobacillus*, *Bifidobacterium*, and *Streptococcus thermophilus*, modulate adaptive immunity by reducing Th17 and promoting Treg, resulting in a decrease in pro-inflammatory cytokines such as IL-1β and TNF-α in patients with TBI (68). Additionally, *Astragalus* flavonoids inhibit EAE by suppressing Th17 and Th1 while enhancing Treg, regulating JAK/STAT pathways (55).

Curcumin, a polyphenol from turmeric, induces tolerogenicity in dendritic cells, reducing Th1, Th2, and Th17 polarization and promoting Treg generation (108, 109). Other compounds, including crocetin, indolepropionic acid, and zinc, also modulate Th17/Treg responses, exerting beneficial effects in hypertension and autoimmune diseases (110–113).

Natural derivatives, including 2′,4′-dihydroxy-2,3-dimethoxychalcone and resveratrol, function as RORγt antagonists, inhibiting Th17 differentiation and alleviating conditions such as colitis and EAE (114, 115). Furthermore, ginsenoside and *Rhodiola rosea* attenuate neuroinflammation in EAE by modulating the Foxp3/RORγt/JAK2/STAT3 axis and restoring the Th17/Treg balance (116–118).

Iron restriction emerges as a novel strategy, as it blocks Th17 differentiation by increasing IL-2 and altering the epigenetic modifications in the IL-17A locus (119). On the other hand, vitamin B5 inhibits glycolysis and the phosphorylation of STAT3, suppressing Th17 pathogenicity in colitis and EAE (120).

Probiotics and fecal microbiota transplants restore the Th17/Treg balance by modulating SCFAs production and improving gut barrier integrity, resulting in protective effects against systemic inflammation and cognitive dysfunction (82, 121–124).

SZB120, a derivative of 3-acetyl-b-boswellic acid, exerts its action by interacting with the α subunit of eukaryotic initiation factor 2 (eIF2α), promoting its phosphorylation at serine 51. This mechanism specifically inhibits Th17 differentiation in murine models, highlighting its potential as a targeted therapy (125). Catalpol, the active component of *Rehmanniae Radix*, regulates Treg-to-Th17 transdifferentiation by upregulating the microRNA let-7g-5p, which reduces STAT3 levels. This effect is particularly relevant in rheumatoid arthritis, where catalpol suppresses both conventional Th17 differentiation and their generation from Tregs, thus modulating the inflammatory response (126).

Artemisinin derivatives, such as TPN10466 and 9,10-anhydrodehydroartemisinin (ADART), have shown efficacy in EAE models. TPN10466 inhibits immune cell migration to the CNS and Th1/Th17 differentiation (127), while ADART reduces demyelination by decreasing inflammatory infiltration and levels of IFN-γ and IL-17A in lymph nodes and the CNS (128). Astragaloside IV, another natural compound, exerts antidepressant effects by modulating the gut microbiota and restoring the Th17/Treg balance, increasing beneficial bacteria such as *Lactobacillus* and *Oscillospira* (129).

Dipsacoside B, derived from Chuan Xu Duan, provides protection against sepsis-associated encephalopathy by preventing Th17 cell infiltration into the brain. Its mechanism involves suppression of the VEGFA/VEGFR2/eNOS pathway, preserving the BBB and reducing neuroinflammation (130). Fungal compounds like the aureane-type sesquiterpenoid tetraketides from *Myrothecium gramineum* also inhibit IL-17A and glycolysis in Th17, attenuating immunopathology in animal models (131).

Berberine, an alkaloid with broad immunomodulatory effects, reduces neuroinflammation in EAE by decreasing pro-inflammatory cytokines (IFN-γ, TNF-α, IL-17) and increasing anti-inflammatory cytokines (IL-4, IL-10, TGF-β). Additionally, it promotes the expansion of Treg and Th2 cells, balancing the immune response in a dose-dependent manner (132). Its effect also extends to other autoimmune diseases by inhibiting critical pathways like STAT and RORγt, as well as modulating the gut microbiota (133).

Ursolic acid inhibits Th17 differentiation via the STAT3/RORγt pathway and blocks its migration by reducing the expression of chemokines (CXCL9/CXCL10) in Schwann cells, without significantly affecting CCL20 (134). 3,3′-Diindolylmethane, an aryl hydrocarbon receptor activator, preserves Treg function in EAE by indirectly reducing Th17 generation and pro-inflammatory cytokines (135). Salidroside improves ischemic brain tolerance by restoring the Th17/Treg balance and suppressing STAT3, thereby attenuating ischemia-reperfusion damage (136). Dietary polyphenols modulate immunity by inhibiting pro-inflammatory subtypes (Th1, Th2, Th17) and promoting Treg, proving useful in diseases such as type 1 diabetes and multiple sclerosis. Their ability to suppress inflammatory cytokines (TNF-α, IL-6) and protect against demyelination makes them promising candidates for nutritional interventions (137).

Natural compounds and probiotics provide a multifaceted approach to regulating the Th17 response, acting on metabolic, transcriptional, and microbial pathways. These findings highlight their potential as adjuncts in managing inflammatory and autoimmune diseases, although further studies are needed to optimize their clinical use.

### 7.2 Pharmacological targets

Th17 play a central role in the pathogenesis of autoimmune and neurodegenerative diseases. Their differentiation and activity are regulated by a complex network of transcriptional, metabolic, and epigenetic factors. In the experimental phase, nuclear receptor agonists, such as peroxisome proliferator-activated receptor gamma (PPARγ) and liver X receptor (LXR), have shown efficacy by inhibiting Th17 differentiation and attenuating pathology in PD models. Additionally, the use of a neutralizing antibody against IL-17A significantly improved symptoms in a rat PD model, suggesting that interrupting this pathway could be therapeutically relevant (9).

The dysregulation of the Th17/Treg balance is a hallmark of many autoimmune diseases. Itaconate, an immunomodulatory metabolite, emerges as a key regulator by inhibiting Th17 differentiation and promoting Treg differentiation through metabolic and epigenetic mechanisms. This compound reduces glycolysis and oxidative phosphorylation in T cells, altering the availability of metabolites like 2-hydroxyglutarate and modifying chromatin accessibility in genes critical for Th17 differentiation, such as RORγt. These changes reduce RORγt binding to the IL-17A promoter, thereby decreasing IL-17A production. The adoptive transfer of itaconate-treated Th17 cells ameliorated EAE, underscoring its potential for clinical application (138).

Another promising strategy is the inhibition of cathepsin B, a protease involved in neuronal death and MS pathology. The inhibitor CA-074 not only protects against ischemia-induced neuronal apoptosis but also reduces RORγT expression in brain tissues of mice with EAE, suggesting a dual effect on neuroprotection and Th17 response regulation (139). On the other hand, phosphoenolpyruvate, a glycolytic metabolite, regulates the Th17 transcriptional program by inhibiting the binding of the JunB/BATF/IRF4 complex to DNA without significantly affecting glycolysis or cell proliferation. This finding opens new pathways to selectively modulate autoimmunity without compromising T cell general function (140).

Bilirubin nanoparticles (BRNPs) have shown efficacy in suppressing EAE by inhibiting antigen-presenting cell maturation and Th17 differentiation without inducing systemic immunosuppression (141). On the other hand, the deficiency of selenoprotein I in T cells alters the metabolic reprogramming required for Th17 activation, reducing their pathogenicity and increasing Treg generation, which provides protection against EAE (142). Finally, activation of PKM2 with compounds such as TEPP-46 inhibits Th17 differentiation, although it may redirect EAE pathology to the brain due to increased GM-CSF (143).

### 7.3 Lifestyle and environmental factors

Lifestyle and environmental factors play a crucial role in modulating the Th17-mediated immune response, influencing the development of inflammatory, metabolic, and neurodegenerative diseases. In overweight and obese children, a significant increase in the percentage of Th17 cells has been observed compared to those with normal weight, and this proportion decreases after weight loss, suggesting a direct relationship between adiposity and Th17 polarization (144, 145). Additionally, the type of physical exercise influences the dynamics of these cells: cardiorespiratory exercise reduces circulating Th17, while resistance exercise increases their mobilization. Regular physical activity also attenuates the increase in Th17 associated with aging, highlighting its modulatory role in adaptive immunity (146).

Diet is another determining factor. A high salt intake promotes the differentiation of pathogenic Th17 cells, linked to inflammatory diseases and hypertension. Excess sodium induces the production of IL-17, which alters endothelial function by reducing nitric oxide synthesis through eNOS phosphorylation, contributing to vascular dysfunction (28, 147–151). In animal models, a diet high in fructose or salt exacerbated hypertension in salt-sensitive rats through the expansion of Th17, while resistant rats showed an anti-inflammatory profile dominated by Treg and IL-10 (152). Furthermore, excessive fat intake leads to a Th17/Treg imbalance in the intestine, compromising the intestinal barrier and worsening anxiety-like behaviors via the AMPK-SIRT1 pathway (153).

Psychological stress and trauma also elevate Th17 levels, which could explain their association with neuropsychiatric disorders such as depression and suicide risk, where premature aging of the immune system and an increase in these cells have been observed (154, 155). Additionally, intestinal dysbiosis, induced by factors such as constipation or antibiotic use, alters the Th17/Treg balance and exacerbates conditions such as EAE or postoperative cognitive decline (156, 157). The intestinal microbiota regulates salt-sensitive hypertension through modulation of the Th17 axis and metabolites such as short-chain fatty acids, which influence intestinal barrier integrity and systemic inflammatory response (158).

Exposure to environmental toxins, such as cigarette smoke, promotes Th17 polarization through dendritic cell activation, an effect that can be mitigated by erythromycin inhibiting the CD40/CD40L pathway (159). On the other hand, high glucose consumption exacerbates autoimmune diseases by inducing Th17 differentiation through the generation of ROS and activation of TGF-β (160, 161).

Interestingly, centenarians present a unique immune profile, with a reduced Th17/Treg ratio and balanced cytokine secretion, suggesting adaptive mechanisms to counteract inflammaging (162). In contrast, in obesity and type 2 diabetes, the Th17/Treg imbalance, driven by cytokines like IL-6 and metabolic pathways such as mTOR, worsens insulin resistance and liver fibrosis (163).

Factors such as diet, exercise, stress, microbiota, and environmental exposure critically modulate the Th17 response, influencing the risk of inflammatory and metabolic diseases. This evidence underscores the importance of personalized lifestyle interventions to restore immune balance and prevent pathologies associated with Th17 dysregulation. **Table 1** summarizes the therapeutic modulation of Th17 cells.

**Table 1:** Therapeutic modulation of Th17 cells.

Therapeutic agent	Mechanism	Disease/model
Curcumol	Polarizing microglia, ↑Nrf2/HO-1, ↓NF-κB, ↓ROS	Cerebral ischemia-reperfusion injury/C57BL/6 mice (33)
Gut microbiota	↓Th17 differentiation	Parkinson disease/review (9)
Huangqi-Guizhi-Wuwu Decoction	T cell differentiation, JAK/STAT	EAE/mice (105)
SCFAs	Regulation of Th17	Perioperative neurocognitive disorders/Wistar rats (106)
Angong Niuhuang Pills	Th17/Treg balance	Atherosclerosis/ApoE-/- Mice (107)
Probiotics	↓Th17, ↑Treg	TBI patients (68)
Astragalus flavonoids	JAK/STAT, ↓Th17, Th1	EAE/mice (55)
Curcumin	↓Th1, Th2, Th17, ↑Treg	Review (108)
Resveratrol	↓RORγt, ↓Th17	Colitis, EAE/mouse, *in vitro* (114, 115)
Ginsesonide Rd, Rhodiola rosea	Foxp3/RORγt/JAK2/STAT3	EAE/EAE mice (116–118)
Iron restriction	↓Th17 differentiation, ↑IL-2	EAE/C57BL/6J mice (119)
Vitamin B5	↓Glycolysis, ↓phosphorylation of STAT3, ↓Th17	Colitis, EAE/cells (120)
SZB120	eIF2α	EAE/C57BL/6 mice (125)
Catalpol	microRNA let-7g-5p, ↓STAT3	Rheumatoid arthritis/mice (126)
Artemisinin	Immune cell migration, Th1/Th17 differentiation	EAE/C57BL/6 mice (127)
Astragaloside IV	Gut microbiota, Th17/Treg balance	Depression/Sprague−Dawley rats (129)
Dipsacoside B	↓VEGFA/VEGFR2/eNOS	Sepsis-associated encephalopathy/C57BL/6 mice (130)
Berberine	↓IFN-γ, TNF-α, IL-17, ↑IL-4, IL-10, TGF-β	EAE/C57BL/6 mice (132)
Ursolic acid	STAT3/RORγt	Th17 cells/rats (134)
Salidroside	Th17/Treg balance, ↓STAT3	Ischemic brain/rats (136)
Dietary polyphenols	↓Th1, Th2, Th17, ↑Treg	Type 1 diabetes, MS/review (137)
Itaconate	↓Th17 differentiation, ↑Treg	EAE/mice (138)
CA-074	↓RORγt	EAE/SJL/J mouse (139)
Phosphoenolpryruvate	JunB/BATF/IRF4	Th17 cells/HEK293 Cells (140)
Bilirubin nanoparticles	Antigen-presenting cell maturation, Th17 differentiation	EAE/mouse (141)
TEPP-46	↓Th17 differentiation	EAE/mouse (143)
Weight loss	↓Th17	Obesity/human (144, 145)
High salt diet	↑Th17, IL-17, ↓nitric oxide	Hypertension/rats (152)
Psychological stress	↑Th17	Depression/mice and patients (154, 155)
Toxins	↑Th17	Chronic obstructive pulmonary disease/mice (159)

### 7.4 Limitations of Th17- and IL-17–targeted therapies

Despite the growing interest in natural compounds, probiotics, and novel pharmacological targets to modulate the Th17 response, several important limitations must be acknowledged. First, most anti-Th17 therapies have been evaluated primarily in animal models or *in vitro* studies, which precludes definitive conclusions regarding their efficacy and safety in humans. Moreover, the heterogeneity of experimental models, the complexity of the immune microenvironment, and the plasticity of Th17 cells hinder the extrapolation of results and the prediction of long-term effects. Microbiome-dependent interventions also exhibit substantial interindividual variability, limiting their standardization. In addition, the lack of longitudinal studies assessing the durability of these effects and their potential risks remains a critical gap, as excessive inhibition of the Th17 pathway could compromise host defense against extracellular pathogens. Therefore, although current findings are promising, well-designed clinical trials are required to validate these strategies in humans before their therapeutic implementation.

## 8 Conclusion

Dysregulation of the Th17/IL-17A response represents a fundamental pathogenic mechanism in neurological and cerebrovascular diseases, promoting neuroinflammation through infiltration into the CNS, microglial activation, and disruption of the blood–brain barrier. Imbalance within the Th17/Treg axis emerges as a central finding, acting both as a driver of pathology and as a potential therapeutic target. Strategies such as IL-17A blockade, RORγt modulation, and restoration of immune homeostasis through pharmacological or natural interventions hold considerable promise in mitigating this inflammatory component. Nevertheless, further studies are required to validate these preclinical findings and ensure their safe and effective translation into clinical practice.

## Glossary

A2aR: A2a receptor
AD: Alzheimer’s disease
ADAM9: A Disintegrin And Metalloproteinase 9
ADART: 9,10-anhydrodehydroartemisinin
AdMSCs: Adipose-derived Mesenchymal Stem Cells
Akt: Protein Kinase B
AMPK: AMP-activated Protein Kinase
APP: Amyloid Precursor Protein
Aβ: amyloid-β
BATF: Basic Leucine Zipper Transcription Factor TF-like
BAX: Bcl2 Associated X Protein
BBB: Blood-Brain Barrier
BCSFB: Cerebrospinal Fluid–Brain Barrier
BRNPs: Bilirubin nanoparticles
CA-074: N-(L-3-trans-propylcarbamoyloxirane-2-carbonyl)-L-isoleucyl-L-proline
cAMP: Cyclic Adenosine Monophosphate
Cbl-b: Casitas B-Lineage Lymphoma proto-oncogene b
CCL2: Chemokine (C-C motif) Ligand 2
CCL20: C-C Motif Chemokine Ligand 20
CCR6: C-C Motif Chemokine Receptor 6
CD4+: Cluster of Differentiation 4 positive
CD40: Cluster of Differentiation 40
CD40L: CD40 Ligand
CD69+: Cluster of Differentiation 69 positive
CD8+: Cluster of Differentiation 8 positive
CK2: Casein Kinase 2
CNS: Central Nervous System
CX4945: Silmitasertib
CXCL1: Chemokine (C-X-C motif) Ligand 1
CXCL10: Chemokine (C-X-C motif) Ligand 10
CXCL2: Chemokine (C-X-C motif) Ligand 2
CXCL8: Chemokine (C-X-C motif) Ligand 8
CXCL9: Chemokine (C-X-C motif) Ligand 9
DNA: Deoxyribonucleic Acid
EAE: Experimental Autoimmune Encephalomyelitis
EGR2: Early Growth Response 2
eIF2α: α Subunit of Eukaryotic Initiation Factor 2
eNOS: Endothelial Nitric Oxide
ERK: Extracellular Signal-Regulated Kinase
FcgR2B: Fc Gamma Receptor 2B
Foxo1: Forkhead box O1
Foxp3: Forkhead box P3
FPR2: Formyl Peptide Receptor 2
GABA: Gamma-Aminobutyric Acid
GATA3: GATA Binding Protein 3
GCN2: General Control Nonderepressible 2
G-CSF: Granulocyte Colony-Stimulating Factor
GM-CSF: Granulocyte-Macrophage Colony-Stimulating Factor
HIF1α: Hypoxia-Inducible Factor 1-αlpha
HO-1: Heme Oxygenase-1
ICAM-1: Intracellular Adhesion Molecule-1
ICH: Intracerebral Hemorrhage
IDO1: Indoleamine 2,3-Dioxygenase 1
IFN-γ: Interferon-γ
IGF-1: Insulin-like Growth Factor 1
IL-10: Interleukin-10
IL-17: Interleukin-17
IL-17A: Interleukin-17A
IL-1β: Interleukin-1β
IL-21: Interleukin-21
IL-23: Interleukin-23
IL-23R: Interleukin-23 Receptor
IL-4: Interleukin-4
IL-6: Interleukin-6
ILC3s: Type 3 Innate Lymphoid Cells
iNOS: inducible Nitric Oxide Synthase
IRF4: Interferon Regulatory Factor 4
ITK: Interleukin-2 (Inducible) T-cell Kinase
JAK: Janus Kinase
JAK2: Janus Kinase 2
JKAP: JNK-associated Phosphatase
JunB: Jun B Proto-Oncogene
LKB1: Liver Kinase B1
LOX-1: Lectin-like Oxidized LDL Receptor-1
LXR: Liver X Receptor
m6A: N⁶-Methyladenosine
MAPK: Mitogen-Activated Protein Kinase
MCP-1: Monocyte Chemoattractant Protein-1
MDSCs: Myeloid-Derived Suppressor Cells
METTL3: Methyltransferase-Like 3
microRNAs: micro Ribonucleic Acids
miR-21: microRNA-21
miR-384: microRNA-384
MMP-3/9: Matrix Metalloproteinase-3/9
MMP-9: Matrix Metalloproteinase-9
MMSE: Mini-Mental State Examination
moDCs: Monocyte-Derived Dendritic Cells
MPTP: Mitochondrial Permeability Transition Pore
MS: Multiple sclerosis
mTOR: Mammalian Target of Rapamycin
mTORC1: Mammalian Target of Rapamycin Complex 1
mtROS: Mitochondrial Reactive Oxygen Species
MyD88: Myeloid Differentiation Primary Response 88
NETs: Neutrophil Extracellular Traps
NF-κB: Nuclear Factor kappa-B
NLRP3: NLR Family Pyrin Domain Containing 3
Nrf2: Nuclear Factor Erythroid 2-Related Factor 2
oxLDL: Oxidized LDL
OXPHOS: Mitochondrial Oxidative Phosphorylation
p38: p38 Mitogen-Activated Protein Kinase
PD: Parkinson’s disease
PI3K: Phosphatidylinositol 3-Kinase
PKA: Protein Kinase A
PKM2: Pyruvate Kinase M2
PPARγ: Peroxisome Proliferator-Activated Receptor gamma
PS1: Presenilin-1, PTEN, Phosphatase and Tensin Homolog
RBP4: Retinol Binding Protein 4
RNA: Ribonucleic Acid
RORC: RAR-related Orphan Receptor C
RORα: RAR-related Orphan Receptor α
RORγt: RAR-related Orphan Receptor gamma t
ROS: Reactive Oxygen Species
SAH: Subarachnoid Hemorrhage
SCFAs: Short-Chain Fatty Acids
SIRT1: Sirtuin 1
Smad3: Sma-and Mothers Against Decapentaplegic Homolog 3
STAT: Signal Transducer and Activator of Transcription
STAT3: Signal Transducer and Activator of Transcription 3
STING: Stimulator of Interferon Genes
TAZ: Transcriptional Co-activator with PDZ-Binding Motif
TBI: Traumatic brain injury
Tc17: T cytotoxic 17
TCF4: Transcription Factor 4
TCR: T cell receptor
Tfh: T Follicular Helper cell
TGF-β: Transforming Growth Factor-β, TGF-β1, Transforming Growth Factor-β1
Th1: T Helper 1 cell
Th17: Helper T cells 17
TLR: Toll-Like Receptor
TLR2: Toll-Like Receptor 2
TLR4: Toll-Like Receptor 4
TLR9: Toll-Like Receptor 9
TLRs: Toll-like receptors
TMAO: Trimethylamine N-Oxide
TNF-α: Tumor Necrosis Factor-α
Treg/Tregs: Regulatory T cells
TRIM21: Tripartite Motif-containing protein 21
UCMSCs: Umbilical Cord-Derived Mesenchymal Stem Cells
USP1: Ubiquitin Specific Peptidase 1
USP19: Ubiquitin Specific Peptidase 19
VCAM-1: Vascular Cell Adhesion Molecule-1
VEGFA: Vascular Endothelial Growth Factor A
VEGFR2: Vascular Endothelial Growth Factor Receptor 2
VSMCs: Vascular Smooth Muscle Cells.
